# The impact of gestational diabetes mellitus on perceived mother-infant bonding: a qualitative study

**DOI:** 10.1080/02646838.2023.2239834

**Published:** 2023-07-26

**Authors:** Madeleine Benton, Jessica Bird, Susan Pawlby, Khalida Ismail

**Affiliations:** Department of Psychological Medicine, King’s College London, London, UK

**Keywords:** Gestational diabetes mellitus, mother-infant bonding, perinatal mental health, pregnancy, postnatal

## Abstract

**Background:**

The prevalence of gestational diabetes mellitus (GDM) is rapidly increasing. It is associated with adverse physical and mental health outcomes for women and their babies. Mother-infant bonding is important for maternal health and infant development, but the effect of GDM on mother-infant bonding has not been examined.

**Objective:**

To explore and describe the impact of GDM on perceived mother infant-bonding in the antenatal and postnatal period.

**Methods:**

Qualitative, individual, semi-structured interviews were conducted with 33 women from diverse backgrounds with current or previous GDM. Data were analysed using reflexive inductive thematic analysis.

**Results:**

Three main themes were generated from the analysis: 1) Concern for baby's health and its impact on bonding; 2) GDM management, the pregnancy experience, and bonding; 3) Continuity and discontinuity of the impact on bonding between the antenatal and postnatal periods.

**Conclusion:**

It was found that GDM can have both positive and negative impacts on perceived mother-infant bonding, which appear to change over the course of the perinatal period. Further observational research is needed to assist in understanding the impact of GDM on mother-infant bonding and the potential mediating effect of mental disorders, including depression.

## Background

### Gestational diabetes mellitus

Gestational diabetes mellitus (GDM) is a form of diabetes that develops in pregnancy. It occurs when hormones produced during pregnancy impact the effectiveness of insulin and results in a build up of glucose in the blood. The prevalence of GDM is increasing, and currently, affects around 15% of pregnancies globally (Wang et al., 2022). Risk factors for GDM include living with overweight or obesity, advanced maternal age, a family history of diabetes, GDM in a previous pregnancy, and belonging to certain ethnic groups (including South Asian, Chinese, African-Caribbean) (Makgoba et al., 2012). GDM is associated with an increased risk of maternal and infant short- and long-term adverse outcomes. One of the most common complications is foetal macrosomia, which can increase birth injury, caesarean section delivery, and shoulder dystocia (Mitanchez et al., 2015). Infants are also at increased risk of neonatal hypoglycaemia, admission to neonatal intensive care, and in rare cases stillbirth (Mitanchez et al., 2015). While GDM usually resolves after delivery, it can have long-lasting health consequences, including a 50% increased risk for GDM recurrence and type 2 diabetes (T2D) in the mother, and future obesity and T2D in the child (Bellamy et al., 2009; Vounzoulaki et al., 2020). Effective management of GDM involves aiming for optimal blood glucose levels. Women with GDM acquire new roles and responsibilities in the pursuit of optimal blood glucose levels, such as self-monitoring blood glucose, diet and exercise modification, highly frequent monitoring by healthcare professionals, and for some women, administering pharmacological treatment including metformin and insulin (Farrar et al., 2017).

### The psychological burden of GDM

This intensive management of GDM is physically burdensome and psychologically demanding and has the potential to change the contextual experience of pregnancy to one that is highly medicalised (Parsons et al., 2018). Increasing literature has highlighted the close link between GDM, mental health difficulties, psychological well-being, and quality of life (Delanerolle et al., 2021; Gilbert et al., 2022; Marchetti et al., 2017). It has been shown that depressive symptoms in early pregnancy are associated with the development of GDM and, further, GDM is a risk factor for perinatal depression (Hinkle et al., 2016). A recent meta-analysis found that women with GDM are twice as likely to experience depression than women without GDM (Wilson et al., 2020). A synthesis of the qualitative evidence of women’s experiences of a GDM diagnosis reported high levels of psychological distress (Craig et al., 2020).

### Mother-infant bonding

The mother-infant relationship is multifactorial and complex. Mother-infant bonding is the connection between the mother and her baby involving cognitions and emotions towards the baby (Bicking Kinsey & Hupcey, 2013). This bond develops progressively, initially during pregnancy, then up to and including the baby’s first year of life (Bicking Kinsey & Hupcey, 2013). Mother-infant bonding is associated with maternal well-being and infant development and critical to developing secure attachment (Le Bas et al., 2020; Tichelman et al., 2019). Impaired mother-infant bonding is associated with maternal depression, delayed child development, and development of psychopathology in adulthood for the child (Joas & Möhler, 2021; Moehler et al., 2006). Risk factors for impaired mother-infant bonding include depression, negative feelings towards pregnancy, and maternal stress symptoms (Bind et al., 2021; Lehnig et al., 2019; Moehler et al., 2006; Nakano et al., 2019). These risk factors are also associated with the experience of GDM, and therefore GDM may present challenges for the pregnant mother in terms of bonding with her baby in pregnancy and postnatally. Studies of other medical conditions, including breast cancer and human immunodeficiency virus, which are also associated with maternal psychological stress, burden, and medicalisation of the pregnancy, have reported impaired mother-infant bonding (Tavares et al., 2018; Willcocks et al., 2016). Several randomised controlled trials have demonstrated that modifying impaired mother-infant bonding using psychological techniques can reduce maternal depressive symptoms after birth and improve developmental outcomes of the baby (Cooper et al., 2009; Holt et al., 2021). However, the role of GDM on mother-infant bonding is not well understood.

### Aim

To explore and describe the impact of GDM on perceived mother infant-bonding in the antenatal and postnatal period.

## Method

### Study design

A qualitative study design using individual interviews was conducted. Ethics committee approval was granted by King’s College London Research Ethics Committee (reference number: HR/DP-21/22–26417).

### Recruitment

Purposeful sampling was used to recruit women with a range of socio-demographic characteristics, aiming for diversity in perinatal stage, parity, and ethnicity (Coyne, 1997). Eligibility criteria were: i) current or previous (within the past 3 years) diagnosis of GDM, ii) at least 18 years of age, and iii) able to read and speak fluent English. Women were recruited using advertisements shared within relevant groups (including support groups for GDM, pregnancy support groups, and pregnancy and postpartum support group for ethnically diverse groups) on social media platforms. The study advertisement highlighted that researcher’s wanted to understand women’s experiences of GDM, and how it impacted on women’s mental health and the relationship with their baby. We did not explicitly use the term ‘bonding’ in order to minimise the risk of inadvertently attracting only participants with extreme negative or positive experiences and to avoid biasing participants’ interview responses. *N* = 176 interested potential participants responded to the research team’s email address as detailed in the study advertisements. Women who responded to the eligibility screening email and who were eligible were provided with further study information and invited to an interview conducted remotely using video-conferencing software, where informed consented was gained prior to the onset of the interview. There was considerable loss of contact with women between expressions of interest to the research and booking interviews with those eligible. From feedback we were able to obtain this was due to 1) women attending multiple medical appointments or being induced earlier than expected for those in the antenatal period and 2) caring responsibilities for those in the postnatal period. Many women in the postnatal period attended the interview with their baby for practical reasons.

### Data collection

The topic guide was piloted with one woman who had GDM. The first author (MB) conducted individual semi-structured interviews; this ensured common questions were asked of each participant but also allowed enough flexibility for the researcher to pursue interesting lines of inquiry pertinent to and raised by individual participants. Example questions include: Can you tell me about your thoughts and feelings when you were diagnosed with GDM? How do you think your diagnosis of GDM impacted on how you felt towards your baby during pregnancy?

Individual interviews were conducted between January 2022 and March 2022 and ranged between 25 and 61 minutes (M time = 39 minutes). Interviews were recorded, transcribed, and anonymised using study numbers.

### Participants

Participants were 33 women aged between 28 years and 42 years of age (*M* age = 33 years). Nine women were in the antenatal period and 24 in the postnatal period at the time of their interview. Eight women had GDM in more than one pregnancy. The key characteristics of the participants are described in Table 1.

**Table 1.** Key characteristics of the participants.

Participant number	Age	Parity	Number of GDM pregnancies	Antenatal or postnatal	Ethnicity*	What does MIB mean to you?
1	41	3	1	Antenatal	White Other	“emotional and physical connection to your baby, that you can kind of start to develop when they’re in the womb”
2	41	1	1	Postnatal	White British	“it’s just about building a relationship”
3	33	1	1	Postnatal	White British	“I thought it would be like this instant, overwhelming surge of love, it felt more gradual than that”
4	28	2	1	Postnatal	White British	“the connection that you feel with your baby before and after birth, and I think it sort of grows throughout their life as well”
5	34	1	1	Postnatal	White British	“how close you feel with your baby, how you sort of instinctively know them”
6	40	1	1	Postnatal	White Other	“your connection with the baby, the emotions”
7	30	2	1	Postnatal	White British	“I think that it’s kind of pre and post-labour … you bond when you’re pregnant … and then post is really important”
8	36	0	1	Antenatal	White British	“at the moment she’s part of me and it feels like every time she moves it feels like a bonding moment”.
9	28	0	1	Antenatal	Japanese	“it’s just having ‘that’ connection”
10	34	1	1	Postnatal	Mixed Asian	“that he is happy with me. I am doing everything I can for him”
11	36	1	1	Postnatal	Asian	“my feelings towards her, I guess our interactions, and I guess just how I felt with having this new little person in my life I guess”
12	35	1	1	Postnatal	White British	“I felt like bonded to her the moment that I saw that positive line on the pregnancy test. I just loved her so much from the moment that happened and the moment that she went into my arms like I was bonded”.
13	33	2	1	Postnatal	White British	“it’s that kind of connection that you feel between each other”
14	28	0	1	Antenatal	White Other	“For me it means just how you are feeling and responding to your baby … and how you feel baby is responding to you … it’s a mutual thing. So even though baby is not out yet … you can interpret reciprocity by certain experiences like their kicks and movements”
15	31	2	1	Antenatal	White British	“I’ve long been of the opinion that when a woman finds out she’s pregnant, that she automatically has a connection”
16	35	2	1	Postnatal	White Other	“happy to hang out with her and do things … caring about her, feeding, not being angry about her keeping me up all night if she’s super hungry or having a bad day”
17	31	1	1	Postnatal	White British	“how much I love my baby.
18	33	1	1	Postnatal	White British	“it means sort of how I feel about my son, whether I’m attached to him”
19	31	1	1	Postnatal	White British	“bonding afterbirth once baby is here and building that bond with a kind of an actual baby rather than just kind of the idea of a baby”
20	42	3	3	Postnatal	Sri Lankan	“it’s just the connection between a mother and her baby”
21	34	2	1	Antenatal	Japanese	“connecting with your baby and feeling connected”
22	28	2	2	Postnatal	White British	“just having time with the child … making sure that they’re happy with you, you’re happy with them. They know that you’re their source of comfort and their protection if they need it”
23	32	1	2	Antenatal	White British	-
24	36	2	2	Postnatal	Indian	“the connection that you have with your child”.
25	32	2	2	Postnatal	White British	“how close you are with your baby”
26	35	2	1	Postnatal	White British	“just being able to feel a connection with your baby and feel that love for your baby”
27	31	2	2	Postnatal	White British	“it’s just sort of your connection with your child and your feelings that you have”
28	41	4	2	Antenatal	White British	“just that initial connection I guess, when you meet your baby finally”
29	38	1	2	Postnatal	Pakistani	Response not provided.
30	37	1	1	Antenatal	Black Caribbean	Response not provided.
31	33	2	2	Postnatal	White British	“in the beginning it’s all about the skin to skin and just being with your baby all the time and feeding”
32	38	1	1	Postnatal	Chinese	“I think it’s spending time with the baby and being happy with the baby”.
33	36	1	1	Postnatal	White British	“it’s about kind of building an attachment with your child and physical contact and … obviously builds for different people in different ways as well”.

*Ethnicity was defined by participants in response to the question: *“Could you tell me the ethnicity with which you identify?”*.

### Data analysis

Transcripts were imported into NVivo V.12 for data management and analysed using inductive reflexive thematic analysis (Braun & Clarke, 2013). The six-phase process of thematic analysis (Braun & Clarke, 2006) was followed, which entails data familiarisation, generating initial codes, generating and then reviewing themes, and defining and offering names for those themes. The first author completed the initial coding, developed the themes and discussed and refined them with all authors. Sample size sufficiency, data adequacy and theme saturation were assessed during the analysis process using existing models of thematic concordance and data quality (Guest et al., 2006; Vasileiou et al., 2018). This meant data were fully saturated when common themes could be found across the dataset, with no new themes being generated by the time the last transcript was analysed.

## Results

The analysis generated three themes. These themes are illustrated by quotes from the women’s interviews.

### Theme 1. Concern for baby’s health and its impact on bonding

Women described varying levels of fear and worry for their baby after learning they had GDM. This was primarily centred around fear of their baby being large for gestational age, shoulder dystocia, and stillbirth.

“My concern was how big he was going to be because they [healthcare professionals] kept telling me how massive my baby was going to be and it was gonna be huge and a monster.” (Interview 18, postnatal)

Some women were reluctant to develop a relationship with the baby until they had greater certainty of their baby’s health and survival. Other women described not wanting to share the news of their pregnancy with family or friends due to this worry and fear.

“It was definitely a draining experience … all the conversations that I had with my consultants, the whole ‘it could end in stillbirth’ … I felt like I didn’t want to … get over excited during that period ‘cause I just felt it was so uncertain throughout the whole thing, even if it was on track, there was still that part of me that was like this could change in an instant.” (Interview 20, postnatal)

Several multiparous women described increased worry for their baby, fear of stillbirth, and reduced enjoyment of their pregnancy when comparing their GDM to non-GDM pregnancy experiences. Women described feelings of guilt when comparing the different pregnancy experiences and the impact of this on their perceived bonding.

“I feel really guilty saying it … during pregnancy I felt quite disconnected from him … I found out about the gestational diabetes, and I felt like I couldn’t grow my bond anymore because I … convinced myself that I was going to lose him at the end. So I … switched off and I didn’t feel any connection with him at all. I would feel him kicking and just be like ‘oh sit still’, whereas with my other baby, the kicking was like ‘oh she’s kicking again’ … I felt guilty because I was aware of the differences … I never took time to sit and like try and enjoy it.” (Interview 4, postnatal)

### Theme 2: GDM management, the pregnancy experience, and bonding

Many women described an increased focus on, in some cases anxiety around, their pregnancy and baby as a result of intensive medical management involved with GDM including, diet modification, additional scans and constant monitoring of blood glucose, often described as ‘never ending’. For some women this increased feelings of protection for their baby or a ‘special’ ‘protective’ bond.

“because I was being scanned so often, because I was having to think much more consciously about being pregnant and what it meant for the baby… I almost bonded with her more. I was just so much more aware of it, whereas my first [pregnancy without GDM] I just kind of got on with life. Yes, I was pregnant but also I wasn’t having to think about … everything I did every day, everything I ate every day meant that I could potentially be harming her.” (Interview 26, postnatal)

Other women felt the opposite, namely, the focus on GDM management and the associated worry took away time to bond with their baby during the pregnancy and adversely impacted their overall enjoyment of the pregnancy.

“I think the concern about making sure my baby was as healthy as possible overrode … enjoying that time, that bonding. I think that was definitely a shadow throughout all three of the [GDM] pregnancies, no matter what. It was almost. Let’s get to the next appointment and see what my scans look like and what my sugars look like.” (Interview 20, postnatal)

Some women described increased efforts to bond with their baby during pregnancy to compensate for the potential perceived impact GDM may have.

“If anything, I’m probably pushing for more of a bond with them and I’m worrying about them and thinking about them more … anything I put in my body is gonna affect the baby.” (Interview 9, antenatal)

Some multiparous women described being more concerned about bonding with their baby in the GDM pregnancy than previous non-GDM pregnancies.

“I did so much more that pregnancy … trying to talk to her … . ‘we’re gonna get through this, it’s gonna be fine’… I thought that it would impact things when she was born too, so I was trying hard.” (Interview 24, postnatal)

#### Feeling disconnected from baby

Some women described disconnect, tension, and frustration towards their baby in relation to managing their GDM and that their baby wasn’t ‘working with them’ particularly when they did not feel in control of their GDM management.

“I definitely at points felt quite a lot of frustration towards him. Just cause of the diabetes thing which obviously is ridiculous. It’s not his fault … but I couldn’t really separate the idea of…this is what the pregnancy has given me. Thankfully that went away after he was born.” (Interview 19, postnatal)

One multiparous woman compared her feelings towards her baby in the GDM pregnancy to her previous pregnancies without GDM, describing feeling like she and her baby were two separate entities:

“I felt as though it separated us … I know a child is not responsible, but it made us two separate entities instead of one being, so something was going on inside of me and he’s [baby] … apparently stacking on this extra weight, and it’s kind of like ‘help me out here’ … it kind of created a sense of tension … it made me feel as though, well I can control myself, but ‘I can’t control what you just keep on taking and doing’. So it made me feel like two people rather than, I’d always felt like one kind of connected unit before [referring to pregnancies with no GDM]”. (Interview 1, antenatal)

### Theme 3: Continuity and discontinuity of the impact on bonding between the antenatal and postnatal periods

The impact of GDM postpartum on mother-infant bonding was varied. Many described relief after birth that they no longer had GDM, while others described that it was often difficult to ‘switch off’ due to significant lifestyle changes made during pregnancy, knowledge of the long-term consequences of GDM, and impact on their overall pregnancy experience. Several women described, continued worry for their baby, although not to the extent experienced in pregnancy. Worry centred around the child’s health such as the increased risk of T2D and obesity. For some women, feelings of guilt surrounding the GDM diagnosis continued into the postpartum period.

“There are definitely some feelings of guilt that I’ve brought him into the world with this already heightened risk, which no one wants a diabetes diagnosis, so I feel bad”. (Interview 19, postnatal)

Women described considerable diet modification to manage GDM during pregnancy, such as food restrictions, and carbohydrate counting, which some believe impacted thoughts and behaviours around postpartum feeding practices and how they felt about their baby and food consumption.

“Even beyond the pregnancy, I am very conscious of what I eat. I think that will stay with me forever. Because it’s so ingrained in me now and even like what I feed my children, I’m thinking, ‘oh gosh, are they having too many carbohydrates?’. You know they’re having porridge with banana and then an apple on the side and that’s just pure carbohydrates like it just in my mind.” (Interview 26, postnatal)

For some multiparous women, they discussed how this wasn’t something they had experienced with their other children from non-GDM pregnancies.

“I think it’s affected how I view his [baby] relationship with food because he’s higher risk [of T2D] as well … I suddenly sit there and go gosh, is his weight okay … have I got overweight children, am I overfeeding … being detrimental to his future health … I don’t want him to get diabetes … whereas again I was probably more relaxed with my first”. (Interview 13, postnatal)

Almost all women in the postnatal period felt they had a positive bond with their baby. This was particularly highlighted by women who were initially worried about how they were bonding with their baby in pregnancy or who discussed potentially impaired bonding.

“As soon as he arrived, completely in love with him, which … I didn’t expect a bond this fast… I think now he’s here I can separate the diabetes and him quite well.” (Interview 19, postnatal)

For some women, feelings of protection towards their infants from the GDM pregnancy continued postnatally, with some women feeling more protective of this infant compared to their other children from non-GDM pregnancies.

“It [GDM] actually affected my relationship with the baby [baby 2 – GDM pregnancy] … it made me more protective of her and I feel closer to her … whether or not that’s because the pregnancy was tougher? I feel more of a bond to [baby 2 – GDM pregnancy] because it’s almost like we got through it together … it’s affected it in … a positive way compared with the bond that I have with [baby 1 – No GDM pregnancy]”. (Interview 25, postnatal)

## Discussion

GDM had wide-ranging impacts on perceived mother-infant bonding. Women’s experiences varied by gestational stage (i.e. antenatal and postnatal), history of previous GDM, and parity. Women described fear and worry for their baby after receiving a GDM diagnosis, an observation concordant with previous studies (Craig et al., 2020; Martis et al., 2018). Fear was primarily centred around the baby’s growth, obstetric complications, and stillbirth. Some women were reluctant to develop a relationship with the baby until they had greater certainty of the baby’s health and survival and did not want to share the news of their pregnancy with the family or friends due to this worry and fear for baby, potentially impacting the availability of social support for women. The impact of fear and worry on reduced emotional bonding with baby aligns with previous studies highlighting that distress in pregnancy is associated with lower mother-infant bonding (Göbel et al., 2018; Kingston et al., 2012). Further, other research has shown that threats to infant health (e.g. through premature delivery or increased risk of disease) might be associated with disruptions in mother-infant bonding (Willcocks et al., 2016). The importance of effective communication between maternity staff and women is well established and has been highlighted by women as a means to avoid unnecessary anxiety, misunderstandings and incorrect expectations (National Insitute for Health and Care Excellence NICE, 2017; Rowe et al., 2002). Our findings raise the question of how a women’s right to have accurate information about the risks associated with GDM (e.g. macrosomia) can be balanced against the potential emotional risks. Evidence of what contributes to ‘effective communication’ remains undefined despite being promoted as a core skill for maternity care staff (Chang et al., 2018). Further research is therefore needed surrounding the communication of GDM, preferences of women, and sensitive information-giving practices.

Previous qualitative research conducted globally (i.e. in Australia, South Africa, UK) has highlighted psychological distress associated with GDM and its management (e.g. additional growth scans and blood glucose monitoring) (Bandyopadhyay, 2021; Muhwava et al., 2020; Parsons et al., 2018). In this study, many women reflected on intensive medical management during pregnancy, which for some resulted in heightened sensitivity to their baby. This level of sensitivity was initially prompted by GDM worry and management, but for some women it brought feelings of a closer connection with the baby. For other women it had the opposite effect, where increased surveillance led to a focus on ‘glucose numbers’, feeling distracted and stressed which in turn impacted their perceived ability and time to bond during pregnancy. Interestingly, in studies of other medical conditions (e.g. HIV) women reported that anxiety about their condition in pregnancy provided early challenges to bonding but it also contributed to enhanced maternal monitoring and a strong desire for infant protection, contributing to stronger subsequent bonding (Willcocks et al., 2016). Several women in this study described an increased effort to bond with their baby during pregnancy to compensate for the perceived potential adverse impact of GDM. Feelings of frustration and disconnect towards the baby in managing GDM was described by women, mainly when blood glucose was challenging to manage or baby was gaining more weight than desired by health care professionals.

While other studies have reported guilt and shame after the initial diagnosis of GDM (Craig et al., 2020; Martis et al., 2018; Parsons et al., 2018), our study found a legacy effect in the postpartum period. While almost all postnatal women described a positive bond with their baby, which was particularly highlighted by women who were initially worried or felt that GDM had impacted adversely on bonding in pregnancy, some women described tension and worry around infant feeding and baby’s risk of T2D and obesity in later life. To our knowledge, there is a dearth of studies investigating the association between GDM and feeding behaviours of infants of GDM mothers (Parsons et al., 2018). However, the importance of infant feeding behaviours on long-term health outcomes for mother and child (Horta et al., 2023; Victora et al., 2016) is well established and therefore, this is critical to examine in future GDM studies.

## Strengths and limitations

A major strength was the diversity in the sample in terms of age, parity, gestational stage, ethnicity, and previous GDM experience. Recruitment and data collection online allowed for the inclusion of women across England. The use of qualitative methodology with semi-structured interviews, allowing women to speak freely without being restricted to preassigned ideas concerning their beliefs and feelings about the topics discussed. The large number (*n* = 176) of women expressing interest in participating in the study is noteworthy, highlighting the importance of this research for women. One limitation is that the sample was self-selecting in response to an advertisement. Therefore, women who may have been very distressed during pregnancy may have been more motivated to participate than less distressed women. Furthermore, self-selection could have also affected our findings in that nearly all women reported positive bonding postpartum, and women who may have impaired postnatal bonding may find it more difficult to discuss.

## Importance of this research

A greater understanding of the relationship between GDM and mother-infant bonding has widespread implications for the public, women with GDM, and their children. This study raises the question of the extent to which health care professionals are aware of the wide-ranging, mostly distressing psychological experiences of GDM. In addition, what is the most effective method for supporting these women to help improve the quality of the mother-infant bond and overall perinatal experience. Further observational research is needed to increase understanding of the impact GDM has on mother-infant bonding and the potential mediating effect of mental health symptomology, including depression, on the relationship.

## Conclusion

This study highlights both the both positive and negative impacts of GDM on perceived mother-infant bonding. It provides a starting point for the design of future research studies in order to further understand the impact of GDM on mother-infant bonding, the underlying contextual factors, and the mechanisms of the relationship.
